# Insight of Metal Ions in Enzymatic Synthesis of Levan: The Metal-Binding Loop

**DOI:** 10.4014/jmb.2411.11030

**Published:** 2025-02-10

**Authors:** Hyunjun Ko, Minsik Kang, Bong Hyun Sung, Mi-Jin Kim, Jung-Hoon Sohn, Jung-Hoon Bae

**Affiliations:** 1 Synthetic Biology Research Center, Korea Research Institute of Bioscience and Biotechnology (KRIBB), Daejeon 34141, Republic of Korea; 2 Department of Forest Biomaterials Engineering, College of Forest and Environmental Sciences, Kangwon National University, Chuncheon 24341, Republic of Korea; 3 School of Biotechnology, Korea University of Science and Technology (UST), Daejeon 34141, Republic of Korea; 4 Cellapy Bio Inc., Daejeon 34141, Republic of Korea

**Keywords:** Levan, levansucrase, metal ion, metal-binding loop

## Abstract

Levan is a fructose polymer with unique physicochemical properties and physiological activities, making it useful in various industries. It is biologically synthesized from sucrose by the enzyme levansucrase, which does not require metal ions for its fructosylation activity. However, during the molecular characterization of a *Pseudomonas chlororaphis* levansucrase (PcLscA), it was observed that several divalent metal ions stimulate fructosylation activity by over 2.5-fold. To understand the structural basis of this action, a 3D model of PcLscA was constructed using the artificial intelligence-based modeling software (RoseTTAFold). This analysis revealed a novel metal-binding loop motif. Interestingly, this newly identified loop motif was also found in six known fructansucrase crystal structures. Furthermore, metal-stimulated levan synthesis was confirmed in the other two levansucrases. By identifying this loop motif, this study suggests an efficient approach for the enzymatic synthesis of levan, which traditionally is not known to require metal ions as activators.

## Introduction

Levan, a β-2,6-linked fructose polysaccharide, is a natural carbohydrate polymer with well-known various health benefits, including prebiotic, anti-inflammatory, antioxidant, antitumor, and cell proliferation benefits [1]. Due to these advantages, levan has been widely utilized in various fields, particularly in the food industry, where it serves as prebiotics, functional sweeteners, and food additives for emulsifying and gelling agents [2]. Levan is biologically synthesized from sucrose by the enzyme, levansucrase (LSRase, EC 2.4.1.10), and the levansucrases have been discovered in various bacteria [3]. The molecular structure of levan, such as the degree of polymerization (DP; also referred to as molecular weight, MW) and degree of branching (DB), which determine levan’s physicochemical properties, is altered by levan-producing bacteria [4]. To date, the relationship between levan’s molecular structure and biological function remains unclear; however, high DP and low DB levan produced in Gram-negative bacteria has been extensively studied in biomedical applications such as anti-cancer materials [5, 6], anti-obesity material [7], drug delivery systems [8-10], and wound-healing matrices [11, 12].

*Pseudomonas chlororaphis* is a gram-negative bacteria commonly used in crop cultivation as a soil inoculant for biological control of pathogenic microbes, insects, and nematodes [13, 14]. *P. chlororaphis* interacts with crops through root colonization via a levan-containing extracellular matrix [15]. In this regard, an LSRase from *P. chlororaphis* subsp. *aurantiaca* (PcLscA) was identified and characterized as an enzyme synthesizing high DP and low DB levan [16, 17]; however, its molecular characterization remains unexplored.

Glycoside hydrolases, including LSRase, do not require metal ions as a cofactor [18]. Nonetheless, metal ions such as calcium and ferrous have been suggested as factors for LSRase stability or secretion [19, 20]. Based on the structural analysis, a weak calcium binding site was predicted in *Bacillus subtilis* LSRase [18], and the LSRase’s hydrolysis and fructosylation activities were stimulated by ferrous and manganese ions, respectively [21, 22]. However, a structural explanation of metal ion effects on LSRase activity remains insufficient.

Because the known crystal structures of LSRases are substantially conserved regardless of Gram staining-based classification, we hypothesized that the PcLscA could also have a metal-binding motif affecting catalytic activity. To answer our question, we investigated the effects of metal ions on PcLscA hydrolysis and fructosylation activities, and a model structure of the enzyme was constructed using artificial intelligence-based modeling software (RoseTTAFold) [23]. We discovered a novel metal-binding loop motif through *in silico* prediction and subsequent molecular experiments after aligning the modeled structure with three known crystal structures of bacterial LSRases.

## Materials and Methods

### Strains, Chemicals, and Medium

*P. chlororaphis* type strain KCTC No. 52142 (identical to ATCC13985) was purchased from Korea Collection for Type Cultures (Republic of Korea). *Escherichia coli* DH5α [F^–^ *lac*ZΔM15 *hsd*R17(r– m–) *gyrA*36] and BL21(DE3)[F^– ^ampT *dcm* *hsd*Sβ (rβ– mβ–) *gal*l (DE3)] were employed for genetic manipulation and expression of the target proteins, respectively. The genomic DNA of *P. chlororaphis* was extracted using a Quick-DNA miniprep Kit (Zymo Research Corp., USA). Q5 DNA polymerase, enterokinase, and restriction endonucleases were purchased from New England Biolabs (USA). The In-Fusion HD cloning kit was purchased from Clontech Laboratories (USA). The prepared DNA was purified using Wizard SV Gel and the PCR Clean-Up system (Promega, USA). The NucleoSpin Plasmid Mini Kit was purchased from Macherey-Nagel (Germany). Standard levan was purchased from Cellapy Bio (Republic of Korea). All other chemicals were purchased from Sigma-Aldrich (USA). *P. chlororaphis* was cultured in Luria–Bertani medium (LB), and the *E. coli* transformants were cultured in LB containing 100 mg/ml ampicillin.

### Cloning, Expression, and Characterization of PcLscA

The completed genome sequence of *P. chlororaphis* from the NCBI database (genomic sequence No. NZ_CP061079.1; gene ID 61652585) was used as the reference. The recombinant PcLscA expression vector was constructed using the pET21b vector by amplifying the PcLscA gene from *P. chlororaphis* 52142 genomic DNA using a primer set (forward-1 5'-TAACTTTAAGAAGGAGATATACATATGAAAAGCAACACTGAA-3'/reverse-1 5'-GTGGTGGTGGTGGTGCTCGAGCTTGAGCGTTACATC-3'). The amplified fragment was cloned into the *Nde*I/*Xho*I site of the pET21b vector using the In-Fusion HD cloning kit. The sequence of the recombinant vector was confirmed (Bionics, Republic of Korea) and introduced into BL21 (DE3).

The transformant was cultured in a 250 ml Erlenmeyer flask containing 50 ml of LB at 37°C at 180 rpm until the optical density (OD) at 600 nm was 0.4–0.6. To induce protein expression, isopropyl β-D-thiogalactopyranoside (IPTG) was added to the culture medium at a final concentration of 0.1 mM and incubated at 18°C for 18 h. After cultivation, the cells were harvested by centrifugation at 12,000 ×*g* for 5 min, reconstituted in 50 ml of 25 mM Tris-HCl buffer (pH 7.5), and disrupted by ultrasonication for 5 min with a 10 s pulse interval on ice. The cell lysate was centrifuged at 12,000 ×*g* for 20 min, and the supernatant and pellet were collected. The supernatant was used as a soluble protein sample, and insoluble protein was obtained from the pellet by adding an equal volume of xTractor buffer following the manufacturer’s instructions (Takara Bio, Japan). To optimize protein production, expression levels according to the temperature (18–37°C) and IPTG concentration (0.01–1 mM) were examined. Proteins expressed with His-tag were purified and desalted using a Profinia protein purification system employing 5/50 ml Bio-scale mini Profinia affinity/desalting cartridges following the manufacturer’s instructions (Bio-Rad, USA). To compare protein expression levels, sodium dodecyl sulfate-polyacrylamide gel electrophoresis (SDS-PAGE)[24] was performed. For western blotting, Anti-His Tag antibody produced in mouse (Sigma-Aldrich) and IRDye 800CW goat anti-mouse IgG (Licor, USA) were used, diluted to 1:2,000 and 1:20,000, respectively. Densitometry analysis was conducted using the Licor Odyssey XF and Image Studio Lite system (Licor).

The optimum condition (temperature, pH, and metal ion effect) for PcLscA levan-forming activity was investigated, as described previously [25]. Briefly, 10 mg of purified PcLscA was added into 1 ml of 50 mM buffers at various pH ranges, and the mixture was incubated at 30°C for 30 min. The optimum temperature was tested for ranges 5–65°C. The effects of metal ions were analyzed by adding various chloride salts of metal ions (1 mM barium, calcium, cobalt, copper, ferrous, mercury, lithium, magnesium, manganese, nickel, and zinc) to the enzyme and substrate solution. To halt the enzyme reaction, 100 ml of 1 M sodium hydroxide was added to the mixture.

PcLscA activities were determined by high-performance liquid chromatography (HPLC) measuring the levels of glucose and fructose after the reaction. Total and hydrolysis activities were calculated using liberated glucose and fructose, respectively, and fructosylation activity was determined by subtracting the hydrolysis activity from the total activity [26].

### Identification of the Metal-Binding Domain

The molecular structure of PcLscA was predicted via RoseTTaFold [23]. Crystal structures of LSRases from Gram-negative bacteria *Erwinia amylovora* (PDB ID: 4D47) and *E. tasmaniensis* (PDB ID: 6FRW) were used as template structures for modeling verification. The template modeling score of the model was calculated using online software (https://zhanggroup.org/TM-align/) [27]. The PcLscA model was aligned using PyMol molecular graphic system version 2.5.1 (Schrödinger, USA) with three known structures containing specific metal ions (PDB ID: 6FRW, 1OYG, and 6M0D). The metal-binding motif candidates were screened by selecting amino acids located within 4 Å of each metal ion. To identify a metal-binding motif, each selected amino acid (D58, D60, and N62 for the A site; F258 and T260 for the B site; V319 and T320 for the C site) were substituted to alanine by overlap extension PCR. For A site, forward-4 5'- ATGCCGTTGCGCGCGCTGGCGGGAGCGATAACCTCCGTC-3'/reverse-4 5'- GACGGAGGTTATCGCTCCCGCCAGCGCGCGCAACGGCAT-3'; For B site, forward-2 5'-AACTCGCGCGCGCAGGCGGCCTGTGTCGGT-3'/ reverse-2 5'-GACACAGGCCGCCTGCGCGCGCGAGTTGCC-3'; for C site, forward-3 5'- TATGCCGACGGTGCGGCGGGGCCAGACGGG-3'/ reverse-3 5'- CCCGTCTGG CCCCGCCGCACCGTCGGCATA-3'. The PcLscA mutants were expressed, purified, and used for metal ion effect analysis as described above. To identify the metal-binding loop motif in other fructansucrases, crystal structures of four LSRases (PDB ID: 1W18, 6FRW, 6M0D, and 1OYG) and two inulosucrases (PDB ID: 2YFS and 7BJ5) were extracted from PDB and aligned using PyMol software. Additionally, to verify the effect of metal ions on fructosylation, we conducted levan synthesis reactions by adding 1 mM calcium chloride, as described above, using two recombinant LSRases from *Rahnella aquatilis* (RaLsrA) and *Bacillus subtilis* (BsSacB) obtained from previous studies [25, 28].

### Levan Biosynthesis

Levan biosynthesis was conducted using a 5 L bioreactor (BIOCNS, Republic of Korea). The two-liter reaction mixture was incubated for 8 h under optimal conditions (200 g/l sucrose-containing media, 50 mM maleate buffer pH 6, 15°C, 150 rpm, and 40 mg purified PcLscA). The effect of metal ion-induced levan synthesis was compared by adding 1 mM calcium chloride. During incubation, samples were collected every 1 h for profiling. To purify levan, the reactant was diafiltrated with distilled water using a 100 kDa NMWL filter cartridge (Cytiva, USA). The purified levan was freeze-dried and used for molecular weight determination and Fourier-transform infrared spectroscopy (FT-IR). The molecular weight was determined by HPLC, and FT-IR data was analyzed by Chungnam National University’s Center for Research Facilities (Republic of Korea).

### HPLC Analysis

Carbohydrate profiles and quantification were analyzed using the HPLC-RID system (Agilent 1100 series; Agilent, USA) with the Sugar KS-802 column (Showa Denko, Japan). HPLC-grade water was used as a mobile phase with a 0.6 ml/min flow rate. The column and detector were maintained at 65 and 50°C, respectively. The Sugar KS-806 (Showa Denko) was used to estimate levan molecular weight, the same mobile phase was used at 1 ml/min, and the column was maintained at 50°C. Polysaccharide standards with various molecular weights from 630,000 to 3,755,000 Da were purchased from American Polymer Standards Corp. (Mentor, USA).

## Results

### Expression and Molecular Characterization of PcLscA

The gene encoding PcLscA was cloned and expressed in *E. coli* (DE3) using the pET21b vector. Under the preliminary induction conditions (18°C and 0.1 mM IPTG), SDS-PAGE revealed the predicted molecular weight (47 kDa) of the target protein, which was verified using western blotting (Fig. S1A). *E. coli* is one of the most widely used workhorses for recombinant protein production. However, depending on the characteristics of the target protein and the expression condition, it can form inactive protein aggregates called inclusion bodies. Therefore, it is necessary to establish the optimal expression condition for the target protein. To address this, during the induction phase, we investigated the optimal temperature and concentration of IPTG to optimize the soluble expression level of PcLscA. The ratio of soluble expression and amount of soluble PcLscA were the highest at 25°C (Fig. S1B). Furthermore, the maximum soluble titer of PcLscA was produced by 0.001 mM IPTG, which is the experiment’s lowest concentration (Fig. S1C). The crude protein was purified by immobilized metal affinity chromatography, and PcLscA was eluted with 250 mM imidazole to characterize the optimal conditions and effects of metal ions (Fig. S1D).

**Fig. 1:**
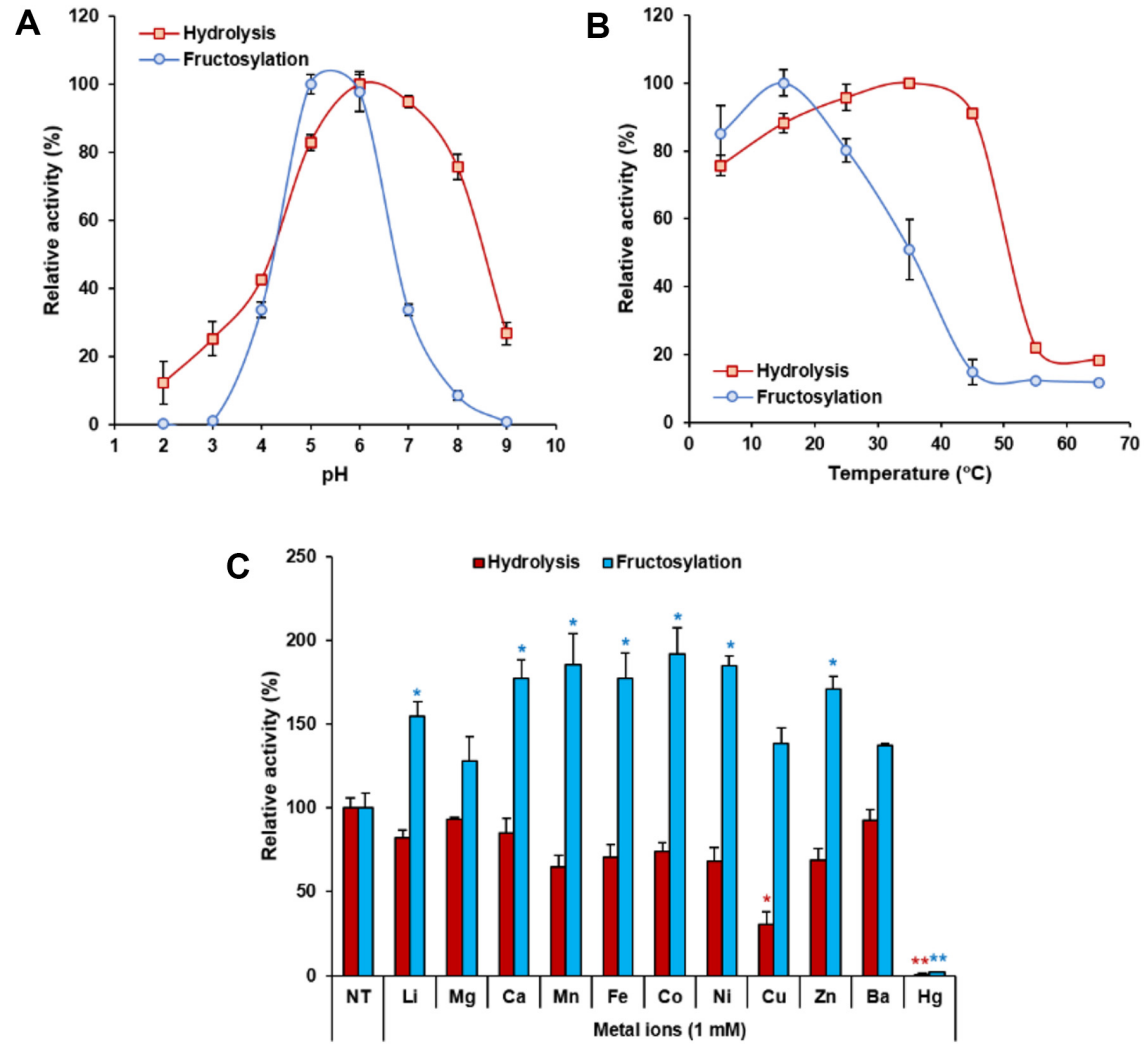
Characterization of PcLscA. The optimum pH (**A**) and temperature (**B**) for PcLscA hydrolysis and fructosylation activities were determined. (**C**) Effects of metal ions (1 mM) on both activities of PcLscA were analyzed. NT, no treatment. All data are represented as the mean ± standard deviation from triplicate experiments. Statistical significance was analyzed by unpaired *t*-test and represented as the number of asterisks: *, *p* < 0.05; **, *p* < 0.01; ns, not significant.

We optimized the two different reactions catalyzed by PcLscA, depending on the acceptor molecule: (1) sucrose hydrolysis (the acceptor molecule was water) and (2) fructosylation (the acceptor molecule was the growing levan). The optimal pH for PcLscA fructosylation (pH 5–6) was slightly acidic than for PcLscA hydrolysis (pH 6–7); however, both reactions were optimal at pH 6 (Fig. 1A). Similar to the general protein stability curve, PcLscA hydrolysis activity increased till 35°C and decreased dramatically after 45C. In contrast, the optimal temperature for PcLscA fructosylation activity was 15°C, with over 80% activity maintained between 5 and 25°C (Fig. 1B). Consequently, levan production can be optimized at 15–35°C.

As discussed in the Introduction section, LSRases do not require metal ions as a cofactor. However, when we investigated the effects of metal ions, PcLscA fructosylation activity was significantly increased (Fig. 1C). Among the tested metal ions, calcium, manganese, ferrous, cobalt, nickel, and zinc increased fructosylation activity approximately 2-fold against the untreated group. PcLscA hydrolysis activity was generally decreased by the addition of metal ions and significantly decreased by copper and mercury ions, which are well-known inhibitors of LSRase hydrolysis activity [29-31].

### Identifying Metal-Binding Site

**Fig. 2:**
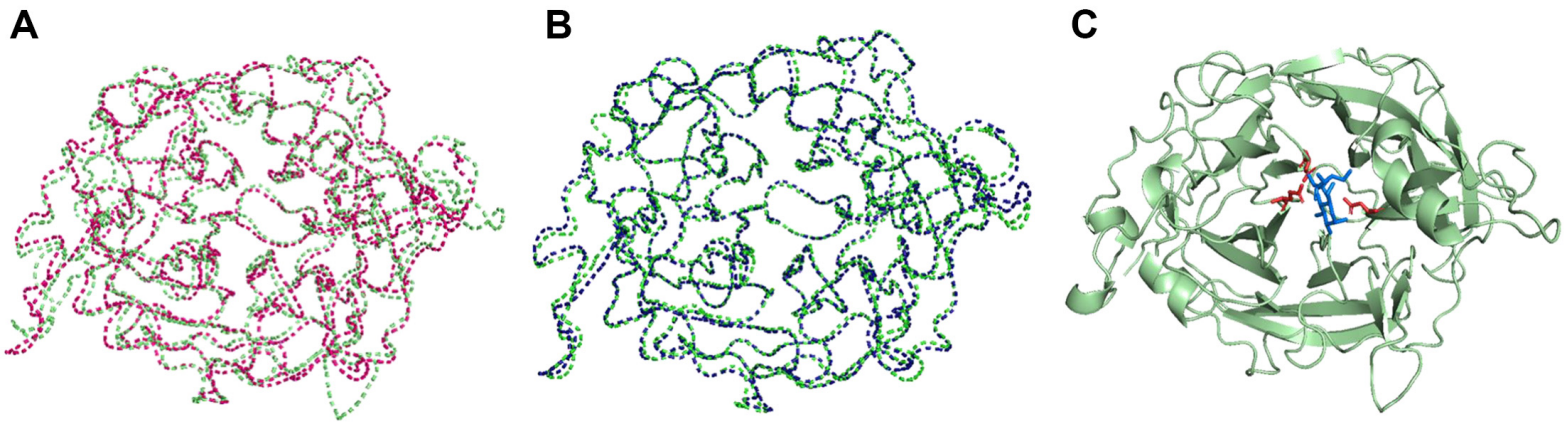
Modeled structure of PcLscA. The template score of the AI-based model of PcLscA (green) and two known LSRase structures (PDB ID: 6FRW, magenta; 4D47, dark blue) were calculated using an online tool (https://zhanggroup.org/TM-align/) and are represented in (**A**) and (**B**), respectively. (**C**) The catalytic triad was represented as red sticks and the substrate (sucrose) was indicated as blue sticks.

Based on the enzyme characterization results, we searched for metal-binding motifs. Because the PcLscA crystal structure was not determined yet, we performed an AI-based modeling (RoseTTAFold) [23]. The confidence score of the model structure was 0.85, and the template model scores of the modeled with 6FRW and 4D47 were 0.97 and 0.96, respectively, indicating high reliability (Fig. 2A and 2B). In the modeled PcLscA, the catalytic triad (D52, D209, and E293) was located in the catalytic pocket within 4 Å with the sucrose molecule (Fig. 2C).

**Fig. 3:**
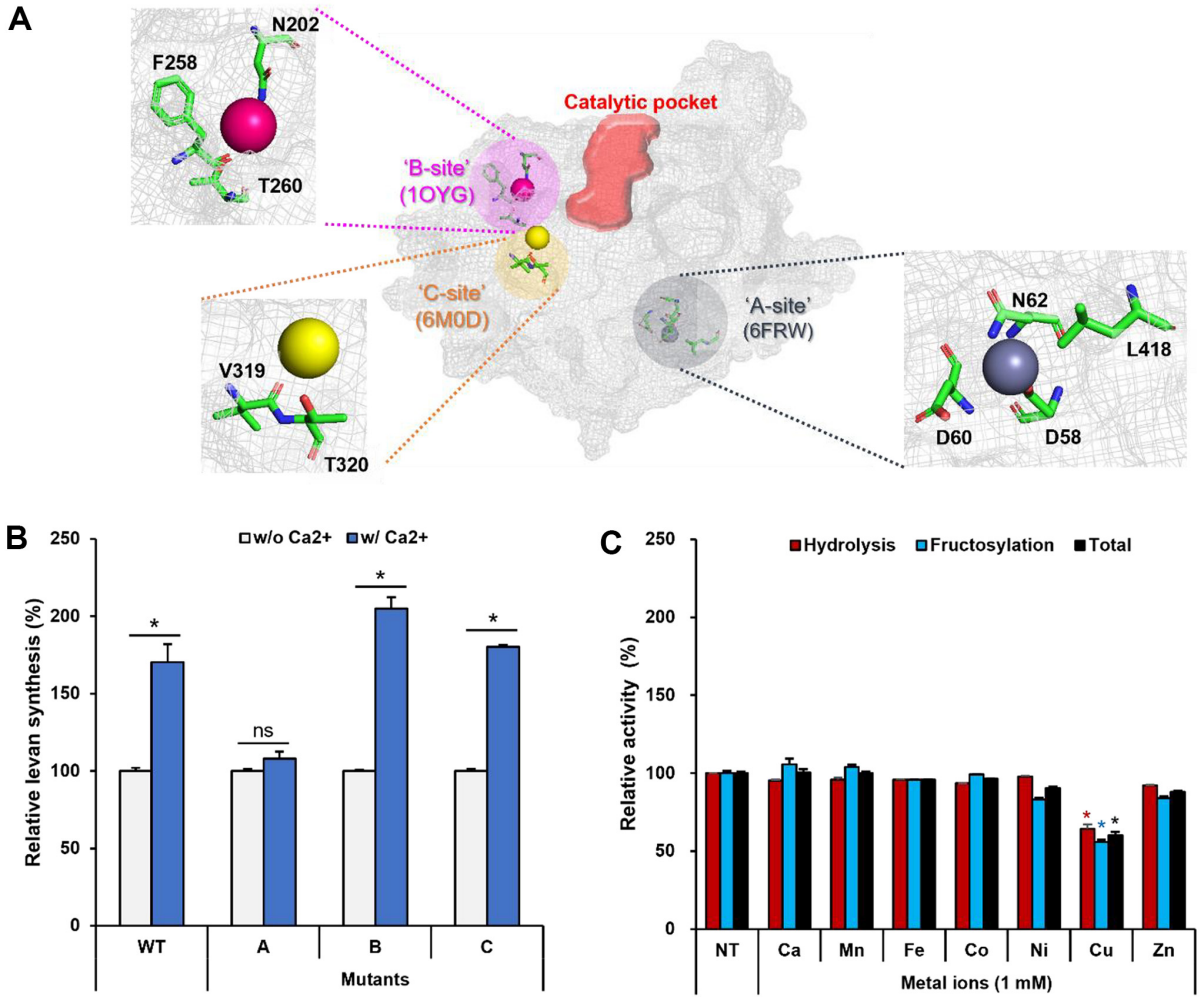
Exploring a metal-binding motif of PcLscA. (**A**) Three metal-binding sites of PcLscA were proposed based on the structural alignment with three known crystal structures of levansucrases (PDB ID: 6FRW, 1OYG, and 6M0D). (**B**) Calcium ion-mediated stimulation of fructosylation activity of the wild-type and three mutants was investigated. (**C**) The effect of the various metal ions for mutant A was tested. WT, wild type; NT, no treatment. All data are represented as the mean ± standard deviation of triplicate experiments. Statistical significance was analyzed by unpaired *t*-test and represented as the number of asterisks: *, *p* < 0.05; ns, not significant.

To locate a metal-binding motif, the model was aligned with three known structures containing specific metal ions (PDB ID: 1OYG for calcium, 6M0D for magnesium, and 6FRW for zinc). After alignment, the amino acids within 4 Å of each metal ion were selected, respectively (Fig. 3A). The A site was a capturing structure of the metal ion by a repeated sequence of charged amino acids (D58, D60, and N62), and the residues highly matched with zinc-binding motifs (D52, D54, and D56) of an LSRase from *Erwinia tasmaniensis* (EtLsc) [32]. The B site was composed of N202, F258, and T260, corresponding to the calcium-binding motif (D241, L308, and N310, respectively) of BsSacB [18]. V319 and T320 from the C site resembled V403 and D404 of an LSRase from *Beijerinckia indica* (BiBftA) [33]. To identify the metal-binding sites of PcLscA, the polar and charged amino acids on each site were substituted with alanine, and the effect of calcium ions on the fructosylation activity of each mutant was tested. Only fructosylation activity of the A-site mutant was not influenced by calcium ions, indicating that it is the metal ion-binding site (Fig. 3B). Further, both hydrolysis and fructosylation activities of the A site mutant were unaffected by various metal ions that increased canonical PcLscA fructosylation by over 2-fold (Fig. 3C). Therefore, we identified that the A site is the metal-binding site of PcLscA.

**Fig. 4:**
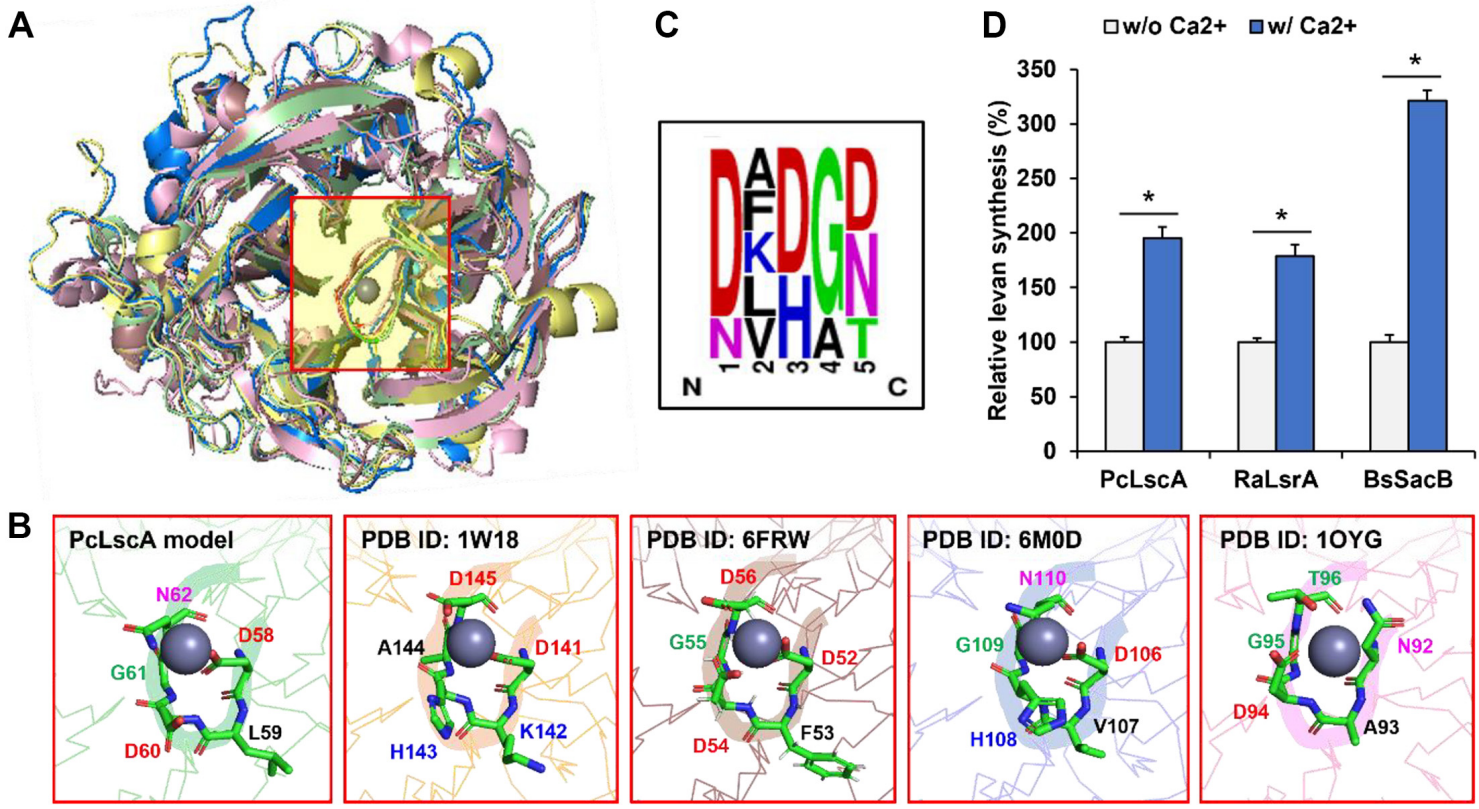
Metal-binding loop motif of levansucrases. (**A**) Certain loop motifs around a metal ion were conserved from a modeled PcLscA and four crystal structures of bacterial levansucrases. (**B**) Identified amino acid sequences of the loop motifs from various levansucrases. (**C**) Sequence logo result of the loop motifs. (**D**) Metal (calcium) ion-mediated stimulation of the fructosylation activity of the three levansucrases. All data are represented as the mean ± standard deviation of triplicate experiments. Statistical significance was analyzed by unpaired *t*-test and represented as the number of asterisks: *, *p* < 0.05; ns, not significant.

The identified metal-binding site comprised a metal ion surrounded by a highly conserved loop structure from the LSRase crystal structures (Fig. 4A and 4B). The sequences of the loops contained five amino acids with two groups: the first, third, and fifth charged amino acids interacted with metal ions, and the second and fourth amino acids were structural elements for the loop (Fig. 4C). The loop structure was identified from all the checked crystal structure of LSRases; thus, we additionally verified the effect of calcium ion on the fructosylation activity of the two LSRases produced in previous studies [25, 28]. The calcium-mediated fructosylation activity was confirmed in all tested LSRases (Fig. 4D).

### Synthesis and Characterization of PcLscA–Levan

**Fig. 5:**
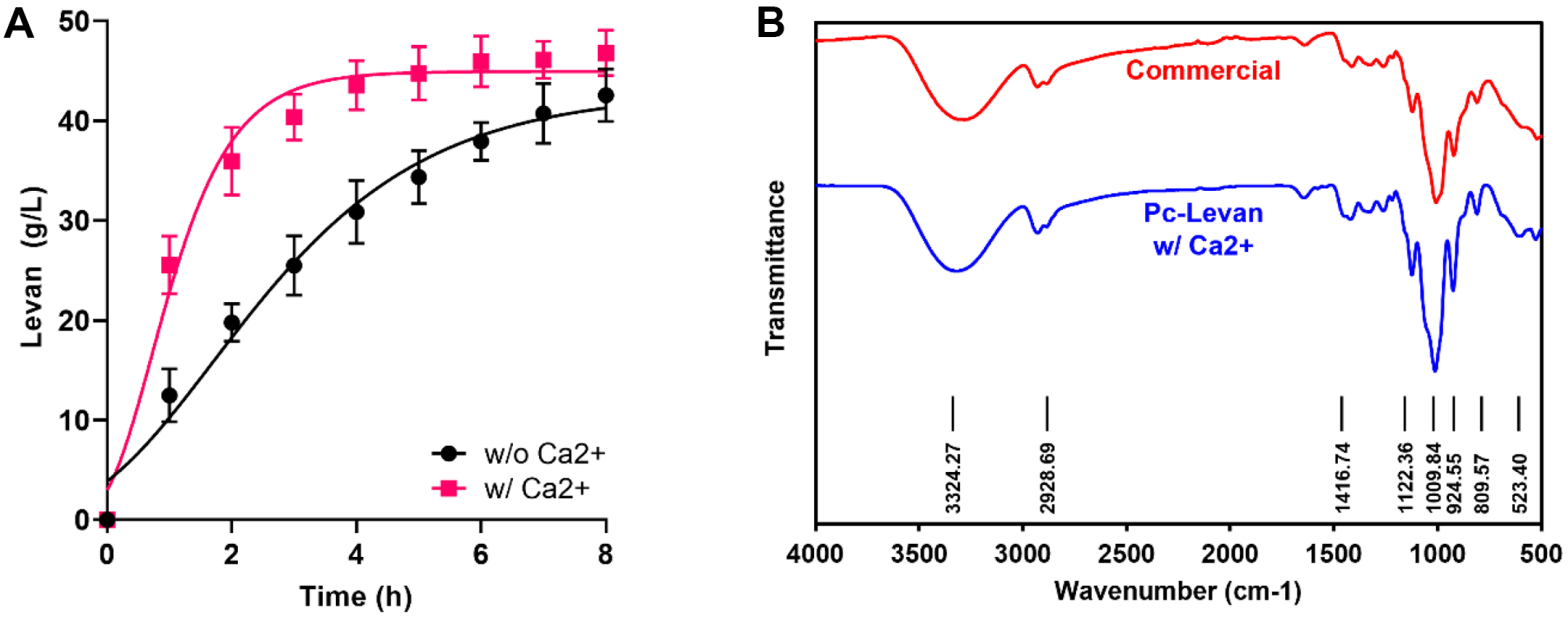
Production and characterization of levan by PcLscA. (**A**) Enzymatic production plots of levan in a 5 L scale bioreactor with or without the addition of a metal ion (1 mM calcium chloride). (**B**) The homogeneity of the commercial and purified levan was analyzed using Fourier-transform infrared spectroscopy (FT-IR).

To compare the levan synthesis rate by a metal ion, the reaction was conducted with or without 1 mM calcium chloride. The levan synthesis plots were represented as the logistic pattern, and the calculated rate constants were 2.75-fold higher in the presence of calcium chloride than in the untreated group (Fig. 5A). The produced levan titer reached 46.8 ± 1.6 g/l from 182.2 ± 0.3 g/l sucrose by adding calcium chloride. To compare the molecular properties of levan synthesized with and without metal ions, we analyzed the molecular weight of the produced levan using HPLC and the functional groups using FT-IR spectrum analysis. The molecular weight of PcLscA–levan was >3,755,000 Da, representing the produced levan in the high molecular weight range, as in the previous study [34]. According to the FT-IR spectra, PcLscA–levan showed broad stretching at approximately 3,300 cm^-1^, indicating the presence of the O-H group and a pyranose fingerprint region at 900-1,100 cm^-1^, consistent with the findings of previous studies [35, 36]. Therefore, we confirmed that adding a metal ion did not affect the structure of the produced levan.

## Discussion

With a growing demand for levan, extensive study has been conducted on the identification, comprehension, and utilization of LSRases. PcLscA was formerly used primarily to produce low-branched and high-molecular-weight levan, serving as an optimal substrate for converting difructose anhydride IV, which is a functional sweetener, without enzymatic characterization [37]. During PcLscA characterization, specific divalent metal ions enhanced PcLscA’s fructosylation activity. This observation is unusual because LSRases are not known to be dependent on metal ions for catalysis. The present study delves into the structural characterization of PcLscA to determine the effects of metal ions on its activity. Among the three potential metal-binding sites predicted by the AI-driven 3D modeling tool, the loop structural A site was identified as the metal-binding site within PcLscA by molecular characterization, and the metal-binding loop motif was proposed. The newly identified metal-binding loop is a compact ensemble of five amino acids featuring a trio of charged amino acids that establishes a potent interaction with the metal ion in conjunction with two structural elements and is intriguingly consistent across different crystal structures of fructansucrases.

Similar to LSRase, inulosucrase (ISRase) synthesizes a type of fructan called inulin (β-2,1-linked) from sucrose, and the ISRase structure is highly conserved with LSRase. Some ISRases display enhanced fructosylation activity in the presence of divalent metal ions, including calcium, ferrous, manganese, nickel, and zinc [38, 39]. Therefore, we additionally performed structural alignment of PcLscA using two known structures of ISRase (PDB ID: 2YFS and 7BJ5) to identify whether the metal-binding loop motif is present. The metal-binding loop motifs were discovered in the structures of ISRases, and the loops were constructed using five amino acids with charged structural patterns, the same as in LSRases (Fig. S2). Glucansucrase (dextransucrase) is another type of carbohydrate polymer synthase-acting sucrose, and some lactic acid bacteria (*Lactobacillus reuteri*, *Leuconostoc citerium*, and *L. mesenteroides*) encode both fructansucrase and glucansucrase genes in their genomes [40]. Additionally, some metal ions activate the catalytic activity of glucansucrases [41-43]. Therefore, we located the metal-binding loop motif in glucansucrases through the structural alignment of PcLscA and three crystal structures of glucansucrases from lactic acid bacteria (PDB ID: 3HZ3, 5LFC, and 3TTO). However, due to poor structural similarity, the metal-binding loop motif was not observed in all tested glucansucrases despite being found in an ISRase from a lactic acid bacterium (*Lactobacillus johnsonii*). Consequently, the metal-binding loop motif may be a unique structure developed during evolution from invertase.

Metal ions play a pivotal role within enzyme systems, wherein certain enzymes necessitate specific metal ions to facilitate their functions. Additionally, metal ions act in catalytic reactions alongside partner enzymes, engaging in electron transfer processes. The stimulatory effect of certain metal ions on levan synthesis by PcLscA was evident in this study, however, the enzyme can produce levan in the absence of these metal ions. Therefore, PcLscA is not classified as a metalloenzyme. Additionally, the identified metal-binding site in PcLscA is located on the opposite side of its catalytic pocket. Considering this, we presume that the stimulated fructosylation of PcLscA by metal ions is an allosteric effect. While allosteric regulation of catalytic activity by metal ions has been observed in diverse enzymes [44], understanding the intricate mechanism within a specific enzyme–ligand complex is difficult due to the dynamic and intricate nature of enzyme systems. To the best of our knowledge, the enhancement of fructosylation activity in PcLscA by metal ions can be elucidated via conformational change. During levan synthesis, the liberated fructose from sucrose is transferred to the developing fructosyl–enzyme intermediate, engaging both hydrolysis and fructosylation activities [45]. The binding of specific metal ions on PcLscA prompts a conformational change more favorable for the growing levan than water as acceptor molecules, accelerating fructosylation activity. To strengthen our hypothesis, advanced experimental techniques such as molecular dynamic simulation and cryogenic electron microscopy would be invaluable.

In conclusion, we successfully elucidated the profound influence of certain divalent metal ions on the fructosylation activity of PcLscA. Our research demonstrated that the addition of calcium chloride effectively stimulates PcLscA, leading to highly efficient levan production without altering its molecular properties. These findings provide novel insights into the allosteric activation of levan, by the metal-binding loop.

## Supplemental Materials

Supplementary data for this paper are available on-line only at http://jmb.or.kr.



## References

[1] Mohd Nadzir M, Nurhayati RW, Idris FN, Nguyen MH (2021). Biomedical applications of bacterial exopolysaccharides: a review. Polymers.

[2] Bersaneti GT, Pan NC, Baldo C, Celligoi MAPC (2018). Co-production of fructooligosaccharides and levan by levansucrase from *Bacillus subtilis* natto with potential application in the food industry. Appl. Biochem. Biotechnol..

[3] Domżał-Kędzia M, Ostrowska M, Lewińska A, Łukaszewicz M (2023). Recent developments and applications of microbial levan, a versatile polysaccharide-based biopolymer. Molecules.

[4] Öner ET, Hernández L, Combie J (2016). Review of levan polysaccharide: from a century of past experiences to future prospects. Biotechnol. Adv..

[5] Calazans GcM, Lima RC, de França FP, Lopes CE (2000). Molecular weight and antitumour activity of *Zymomonas mobilis* levans. Int. J. Biol. Macromol..

[6] Yoo SH, Yoon EJ, Cha J, Lee HG (2004). Antitumor activity of levan polysaccharides from selected microorganisms. Int. J. Biol. Macromol..

[7] Kang SA, Hong KH, Jang KH, Kim SH, Lee KH, Chang BI (2004). Anti-obesity and hypolipidemic effects of dietary levan in high fat diet-induced obese rats. J. Microbiol. Biotechnol..

[8] Sezer AD, Kazak H, Öner ET, Akbuğa J (2011). Levan-based nanocarrier system for peptide and protein drug delivery: optimization and influence of experimental parameters on the nanoparticle characteristics. Carbohydr. Polym..

[9] Sezer AD, Kazak Sarılmışer H, Rayaman E, Çevikbaş A, Öner ET, Akbuğa J (2017). Development and characterization of vancomycinloaded levan-based microparticular system for drug delivery. Pharm. Dev. Technol..

[10] Cinan E, Cesur S, Erginer Haskoylu M, Gunduz O, Toksoy Oner E (2021). Resveratrol-loaded levan nanoparticles produced by electrohydrodynamic atomization technique. Nanomaterials.

[11] Costa RR, Neto AI, Calgeris I, Correia CR, Pinho AC, Fonseca J (2013). Adhesive nanostructured multilayer films using a bacterial exopolysaccharide for biomedical applications. J. Mater. Chem. B..

[12] Bostan MS, Mutlu EC, Kazak H, Keskin SS, Oner ET, Eroglu MS (2014). Comprehensive characterization of chitosan/PEO/levan ternary blend films. Carbohydr. Polym..

[13] Chin-A-Woeng TF, Bloemberg GV, Mulders IH, Dekkers LC, Lugtenberg B (2000). Root colonization by phenazine-1-carboxamideproducing bacterium *Pseudomonas chlororaphis* PCL1391 is essential for biocontrol of tomato foot and root rot. Mol. Plant-Microbe Interact..

[14] Anderson AJ, Kang BR, Kim YC (2017). The Gac/Rsm signaling pathway of a biocontrol bacterium, *Pseudomonas chlororaphis* O6. Res. Plant Dis..

[15] Arrebola E, Tienda S, Vida C, De Vicente A, Cazorla FM (2019). Fitness features involved in the biocontrol interaction of *Pseudomonas chlororaphis* with host plants: the case study of PcPCL1606. Front. Microbiol..

[16] Jang EK, Jang KH, Koh I, Kim IH, Kim SH, Kang SA (2002). Molecular characterization of the levansucrase gene from *Pseudomonas aurantiaca* S-4380 and its expression in *Escherichia coli*. J. Microbiol. Biotechnol..

[17] Jang EK, Kim SH, Kim IH, Kang SA, Koh I, Park YI (2006). High-level production of low-branched levan from *Pseudomonas aurantiaca* S-4380 for the production of di-β-D-Fructofuranose dianhydride IV. J. Microbiol. Biotechnol..

[18] Meng G, Fütterer K (2003). Structural framework of fructosyl transfer in *Bacillus subtilis* levansucrase. Nat. Struct. Mol. Biol..

[19] Petit‐Glatron M, Monteil I, Benyahia F, Chambert R (1990). *Bacillus subtilis* levansucrase: amino acid substitutions at one site affect secretion efficiency and refolding kinetics mediated by metals. Mol. Microbiol..

[20] Petit‐Glatron MF, Grajcar L, Munz A, Chambert R (1993). The contribution of the cell wall to a transmembrane calcium gradient could play a key role in *Bacillus subtilis* protein secretion. Mol. Microbiol..

[21] Belghith KS, Dahech I, Belghith H, Mejdoub H (2012). Microbial production of levansucrase for synthesis of fructooligosaccharides and levan. Int. J. Biol. Macromol..

[22] Szwengiel A, Goderska K, Gumienna MJJoMCBE (2016). Synthesis of ß-(2-6)-linked fructan with a partially purified levansucrase from *Bacillus subtilis*. J. Mol. Catal. B Enzym..

[23] Baek M, DiMaio F, Anishchenko I, Dauparas J, Ovchinnikov S, Lee GR (2021). Accurate prediction of protein structures and interactions using a three-track neural network. Science.

[24] Laemmli UK (1970). Cleavage of structural proteins during the assembly of the head of bacteriophage T4. Nature.

[25] Ko H, Bae JH, Sung BH, Kim MJ, Kim CH, Oh BR (2019). Efficient production of levan using a recombinant yeast *Saccharomyces cerevisiae* hypersecreting a bacterial levansucrase. J. Ind. Microbiol. Biotechnol..

[26] Liu Q, Yu S, Zhang T, Jiang B, Mu W (2017). Efficient biosynthesis of levan from sucrose by a novel levansucrase from *Brenneria goodwinii*. Carbohydr. Polym..

[27] Zhang Y, Skolnick J (2005). TM-align: a protein structure alignment algorithm based on the TM-score. Nucleic Acids Res..

[28] Ko H, Bae JH, Sung BH, Kim MJ, Park SH, Sohn JH (2019). Direct production of difructose anhydride iv from sucrose by cofermentation of recombinant yeasts. Sci. Rep..

[29] Salama BM, Helmy WA, Ragab TI, Ali MM, Taie HA, Esawy MA (2019). Characterization of a new efficient low molecular weight *Bacillus subtilis* NRC 16 levansucrase and its levan. J. Basic Microbiol..

[30] Kirtel O, Menéndez C, Versluys M, Van den Ende W, Hernández L, Toksoy Öner E (2018). Levansucrase from *Halomonas smyrnensis* AAD6T: first halophilic GH-J clan enzyme recombinantly expressed, purified, and characterized. Appl. Microbiol. Biotechnol..

[31] Gao S, Qi X, Hart DJ, Gao H, An Y (2017). Expression and characterization of levansucrase from *Clostridium acetobutylicum*. J. Agr. Food Chem..

[32] Polsinelli I, Caliandro R, Salomone-Stagni M, Demitri N, Rejzek M, Field RA (2019). Comparison of the Levansucrase from the epiphyte Erwinia tasmaniensis vs its homologue from the phytopathogen *Erwinia amylovora*. Int. J. Biol. Macromol..

[33] Tonozuka T, Kitamura J, Nagaya M, Kawai R, Nishikawa A, Hirano K (2020). Crystal structure of a glycoside hydrolase family 68 β-fructosyltransferase from *Beijerinckia indica* subsp. indica in complex with fructose. Biosci. Biotechnol. Biochem..

[34] Jang KH, Jang EK, Kim SH, Kim IH, Kang SA, Koh I (2006). High-level production of low-branched levan from *Pseudomonas aurantiaca* S-4380 for the production of di-β-D-fructofuranose dianhydride IV. J. Microbiol. Biotechnol..

[35] Jathore NR, Bule MV, Tilay AV, Annapure US (2012). Microbial levan from *Pseudomonas fluorescens*: characterization and medium optimization for enhanced production. Food Sci. Biotechnol..

[36] Xavier JR, Ramana KV (2017). Optimization of levan production by cold-active *Bacillus licheniformis* ANT 179 and fructooligosaccharide synthesis by its levansucrase. Appl. Biochem. Biotechnol..

[37] Jang EK, Jang KH, Isaac K, Kim IH, Kim SH, Kang SA (2002). Molecular characterization of the levansucrase gene from *Pseudomonas aurantiaca* S-4380 and its expression in *Escherichia coli*. J. Microbiol. Biotechnol..

[38] Ni D, Zhu Y, Xu W, Bai Y, Zhang T, Mu W (2018). Biosynthesis of inulin from sucrose using inulosucrase from *Lactobacillus gasseri* DSM 20604. Int. J. Biol. Macromol..

[39] Van Hijum S, Van Der Maarel M, Dijkhuizen L (2003). Kinetic properties of an inulosucrase from *Lactobacillus reuteri* 121. FEBS Lett..

[40] van Hijum SA, Kralj S, Ozimek LK, Dijkhuizen L, van Geel-Schutten IG (2006). Structure-function relationships of glucansucrase and fructansucrase enzymes from lactic acid bacteria. Microbiol. Mol. Biol. Rev..

[41] Du R, Qiao X, Wang Y, Zhao B, Han Y, Zhou Z (2019). Determination of glucansucrase encoding gene in *Leuconostoc mesenteroides*. Int. J. Biol. Macromol..

[42] Zhao B, Du R, Wang J, Xu M, Han Y, Han X (2020). Purification and biochemical characterization of a novel glucansucrase from *Leuconostoc citreum* B-2. Biotechnol. Lett..

[43] Miao M, Ma Y, Jiang B, Cui SW, Jin Z, Zhang T (2017). Characterisations of *Lactobacillus reuteri* SK24. 003 glucansucrase: implications for α-gluco-poly-and oligosaccharides biosynthesis. Food Chem..

[44] McCullagh M, Zeczycki TN, Kariyawasam CS, Durie CL, Halkidis K, Fitzkee NC (2024). What is allosteric regulation? Exploring the exceptions that prove the rule!. J. Biol. Chem..

[45] Chambert R, Gonzy‐Treboul G (1976). Levansucrase of *Bacillus subtilis*: characterization of a stabilized fructosyl‐enzyme complex and identification of an aspartyl residue as the binding site of the fructosyl group. Eur. J. Biochem..

