# Supplementary material for: Insight of Metal Ions in Enzymatic Synthesis of Levan: The Metal-Binding Loop
Source: J Microbiol Biotechnol. 2025 Feb 10;35:e2411030. doi: 10.4014/jmb.2411.11030 (PMC11876013; doi:10.4014/jmb.2411.11030)
Supplement: Supplementary file 1 [file jmb-35-e2411030-supple.pdf]

J. Microbiol. Biotechnol. 2025. 35: e2411030
https://doi.org/10.4014/jmb.2411.11030

Supplementary Figures

Insight of metal ions in enzymatic synthesis of levan: the metal-binding loop

Hyunjun Ko[1,2], Minsik Kang[1,3], Bong Hyun Sung[1,3], Mi-Jin Kim[1], Jung-Hoon Sohn[1,3,4,*], and Jung-Hoon Bae[1,*]

[1]Synthetic Biology Research Center, Korea Research Institute of Bioscience and Biotechnology (KRIBB), 125 Gwahak-ro, Yuseong-gu, Daejeon 34141, Republic of Korea

[2]Department of Forest Biomaterials Engineering, College of Forest and Environmental Sciences, Kangwon National University, Chuncheon 24341, Republic of Korea

[3]School of Biotechnology, Korea University of Science and Technology (UST), 125 Gwahak-ro, Yuseong-gu, Daejeon 34141, Republic of Korea

[4]Cellapy Bio Inc., Bio-Venture Center 211, 125 Gwahak-ro, Yuseong-gu, Daejeon 34141, Republic of Korea

***Corresponding authors:** J-H Sohn (sohn4090@kribb.re.kr), J-H Bae (hoon@kribb.re.kr),

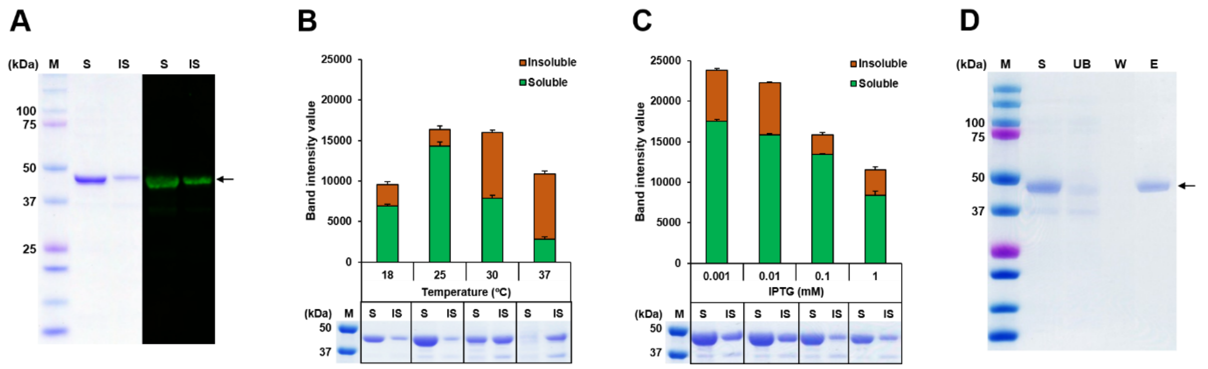

Fig. S1. Heterologous expression and purification of the PcLscA. The recombinant PcLscA was solubly expressed in *E. coli* **(A).** The optimum induction temperature **(B)** and dose of IPTG **(C)** for the production of PcLscA were determined, respectively. The PcLscA was purified via an immobilized metal ion chromatography **(D).** M, molecular parker; S, soluble expression sample; IS, insoluble expression sample: UB, unbound sample; W, washed sample by 20 mM imidazole; E, eluted sample by 250 mM imidazole.

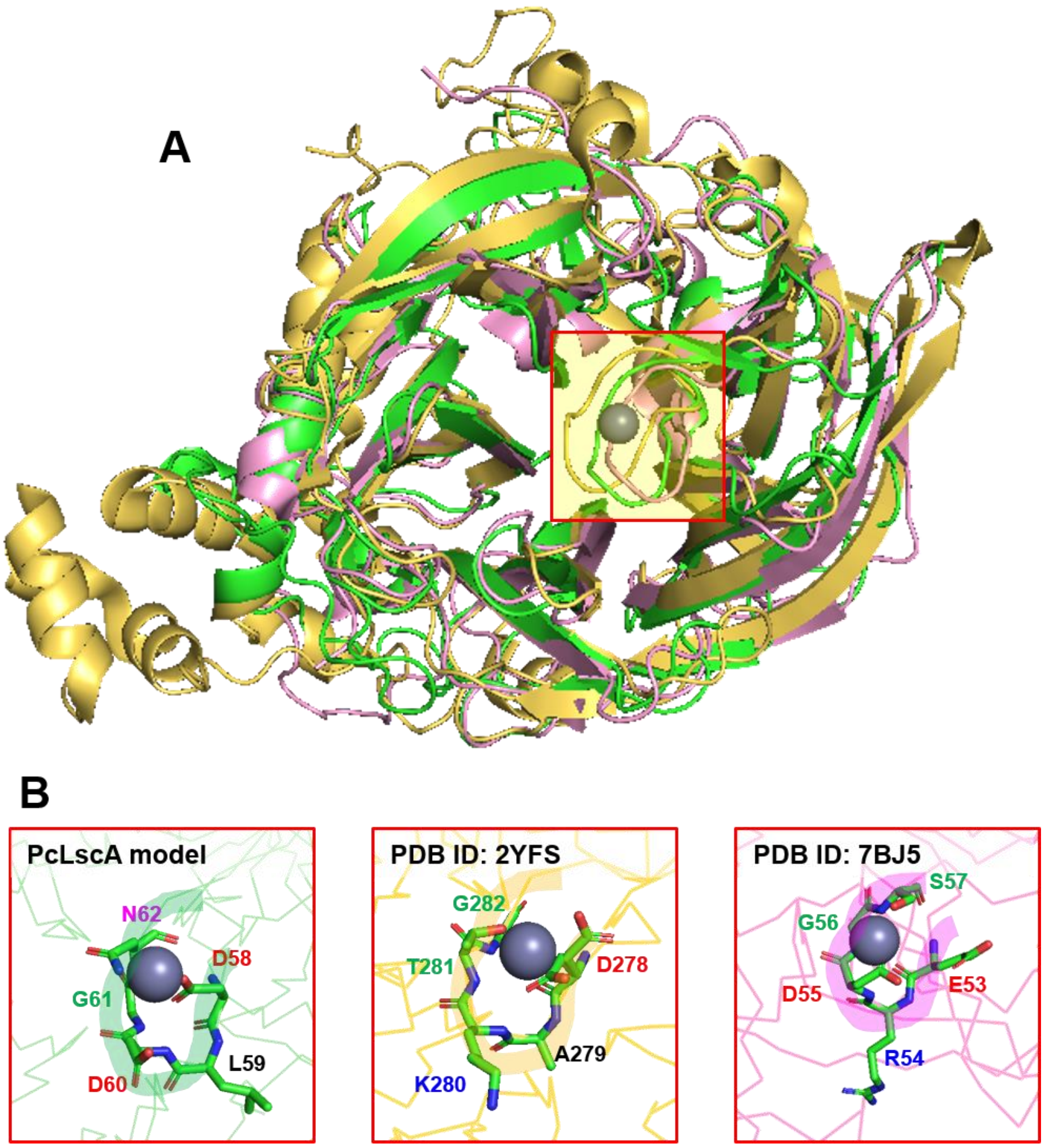

Fig. S2. Metal binding loop of inulosucrases. The metal binding loop motif of inulosucrases were identified by structural alignment analysis **(A).** Identified amino acids sequences of the loop motifs from two inulosucrases were represented on **(B).** (PDB ID: 2YFS and 7BJ5). The modeled structure of PcLscA was used as a template and the metal ion was extracted from PDB ID: 6FRW.
